# IGF-1 Inhibits Apoptosis of Porcine Primary Granulosa Cell by Targeting Degradation of Bim_EL_

**DOI:** 10.3390/ijms20215356

**Published:** 2019-10-28

**Authors:** Ying Han, Shumin Wang, Yingzheng Wang, Shenming Zeng

**Affiliations:** National Engineering Laboratory for Animal Breeding, Key Laboratory of Animal Genetics, Breeding, and Reproduction of the Ministry of Agriculture and Rural Affairs, College of Animal Science and Technology, China Agricultural University, Beijing 100193, China

**Keywords:** IGF-1, Bim_EL_, phosphorylation, apoptosis, autophagy

## Abstract

Insulin-like growth factor-1 (IGF-1) is an intra-ovarian growth factor that plays important endocrine or paracrine roles during ovarian development. IGF-1 affects ovarian function and female fertility through reducing apoptosis of granulosa cells, yet the underlying mechanism remains poorly characterized. Here, we aimed to address these knowledge gaps using porcine primary granulosa cells and examining the anti-apoptotic mechanisms of IGF-1. IGF-1 prevented the granulosa cell from apoptosis, as shown by TUNEL and Annexin V/PI detection, and gained the anti-apoptotic index, the ratio of Bcl-2/Bax. This process was partly mediated by reducing the pro-apoptotic Bim_EL_ (Bcl-2 Interacting Mediator of Cell Death-Extra Long) protein level. Western blotting showed that IGF-1 promoted Bim_EL_ phosphorylation through activating p-ERK1/2, and that the proteasome system was responsible for degradation of phosphorylated Bim_EL_. Meanwhile, IGF-1 enhanced the Beclin1 level and the rate of LC3 II/LC3 I, indicating that autophagy was induced by IGF-1. By blocking the proteolysis processes of both proteasome and autophagy flux with MG132 and chloroquine, respectively, the Bim_EL_ did not reduce and the phosphorylated Bim_EL_ protein accumulated, thereby indicating that both proteasome and autophagy pathways were involved in the degradation of Bim_EL_ stimulated by IGF-1. In conclusion, IGF-1 inhibited porcine primary granulosa cell apoptosis via degradation of pro-apoptotic Bim_EL_. This study is critical for us to further understand the mechanisms of follicular survival and atresia regulated by IGF-1. Moreover, it provides a direction for the treatment of infertility caused by ovarian dysplasia, such as polycystic ovary syndrome and the improvement of assisted reproductive technology.

## 1. Introduction

The insulin-like growth factor (IGF) system includes IGFs, IGF receptors, and multiple binding proteins [1]. In particular, IGF-1 exerts multiple physiologic effects on the systemic vasculature through endocrine, autocrine or paracrine mechanisms [2]. Many studies have reported that IGF-1 widely exists in mammalian ovarian tissues and is indispensable to follicle growth [3,4,5]. For example, the development of ovaries from the mouse deleted IGF-1 gene was incomplete, with the follicles arresting at the pre-antral or early antral stage and the mitotic abilities of granulosa cells presenting as weak [6]. On the contrary, when the second dominant follicle, destined to be atresia, was injected with bioactive IGF-1, it became dominant and eventually showed ovulation in mares [7]. Factually, only a small fraction of mammalian ovarian follicles can ovulate while the vast majority are destined to undergo atresia [8]. However, the underlying mechanisms remain to be deciphered.

IGF-1 is one potential crucial survival factor, besides follicle stimulating hormone (FSH), especially in later antral stages: cultured pre-ovulatory rat follicles have shown suppression of the spontaneous onset of apoptosis treated with IGF-1, which is more effective than insulin [9]. IGF-1 also inhibited apoptosis by 45% in early rat antral follicles [10]. In cattle, one clear detectable difference between dominant follicles selected for development to ovulation and subordinate follicles destined to undergo atresia, was greater free IGF-1 concentrations in the dominant follicles [11]. In situ analysis of apoptotic DNA fragmentation has revealed that granulosa cells in these follicles are the main cell type undergoing apoptosis and granulosa cell apoptosis is widely considered the main reason of ovarian follicular atresia [12,13]. In another study, the levels of IGF-1 and IGF receptor decreased in atretic follicles, while the ratio of apoptotic granulosa cells in atretic follicles was significantly higher, in the healthy follicles of goats [14]. IGF-1 increased proliferation of porcine granulosa cell during folliculogenesis [15]. As a result, the underlying mechanisms of IGF-1 inhibiting apoptosis were explored in granulosa cells and other cells. For example, the PI3K/Akt pathway may be considered as the canonical pathway involved in the inhibition of apoptosis by IGF-1 in many cell styles, including granulosa cells, neurons, and Schwann cells [16,17,18]. The ERK1/2 pathway was also activated by IGF-1 to regulate apoptosis and tissue regeneration in breast cancer cells, neurons, and osteogenic differentiation [19,20,21]. Moreover, by enhancing the progression of the cell cycle and preventing activation of the Fas pathway, IGF-1 has also been shown to play its repression function in granulosa cell apoptosis [11,22,23].

Apoptosis is an essential physiological process to eliminate damaged and infected cells in order to maintain health and the normal functions of organisms [24]. Only a fraction of follicles in the ovary will eventually ovulate, while the vast majority of follicles, present at birth, are destined to undergo apoptosis and atresia [8]. The Bcl-2 protein family is important in regulating cell apoptosis [25]. In particular, Bim_EL_, one important pro-apoptotic Bcl-2 family protein, is identified as the major contributor to apoptosis induced by cytokine deprivation [26]. Cells with low levels of Bim_EL_ have proven to be resistant to death signals, while Bim_EL_ overexpression has facilitated cell death [27]. In granule neurons, IGF-1 signaling via AKT suppressed the transcription of Bim to promote the survival of cerebellar [28]. IGF-1 blocked Bim expression in multiple myeloma through epigenetic and posttranslational mechanisms [29]. In granulosa cells, a previous paper indicated that the follicular atresia and apoptosis of granulosa cells were implicated in increasing pro-apoptotic Bim_EL_ [30,31]. In addition, IGF-1 is one of the survival promoting factors in the ovary. However, the mechanisms of IGF-1 on the granulosa cells’ survival are not fully understood. Therefore, we propose that IGF-1 can inhibit the porcine granulosa cells apoptosis through regulating the pro-apoptotic protein Bim_EL_.

## 2. Results

### 2.1. IGF-1 Prevented Apoptosis and Down-regulated Bim_EL_ Protein Level in Porcine Granulosa Cells

**Figure 1:**
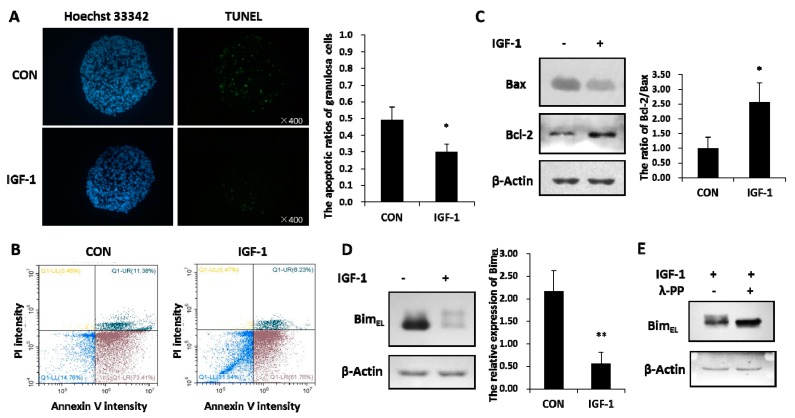
Insulin-like growth factor-1 (IGF-1) inhibited apoptosis and reduced the Bim_EL_ level. (**A**) TUNEL (TdT-mediated dUTP Nick-End Labeling) assay showed the apoptosis percentage of porcine granulosa cells. Cells were cultured in the presence of IGF-1 (50 ng/mL) or absence of IGF-1 (CON) for 24 h. The left panel demonstrates the fluorescence microscopy of DNA fragmentation in cells detected by TUNEL (green). Cell nuclei were stained with Hoechst 33342 (blue) (×400). The right panel is the mortality rate of granulosa cells. (**B**) The image-based analysis of intensity of annexin V-FITC/PI staining showed healthy (blue dots), early apoptotic (brown dots), late apoptotic (bottle-green dots) and dead (yellow dots) cells. Cells were treated with an uncomfortable concentration of hydrochloric acid (0.01 mol/L) 10 min after treatment with or without IGF-1 (50 ng/mL) for 24 h. (**C**) The regulation effect of IGF-1 on the Bcl-2 and Bax protein was demonstrated. (**D**) The expression of Bim_EL_ was downregulated by IGF-1. (**E**) The phosphorylation of Bim_EL_ was induced by IGF-1. The values are expressed as means ± S.D of at least three separate experiments. * *p* < 0.05, ** *p* < 0.01.

Granulosa cells experienced increased apoptosis signals in the untreated group, while IGF-1 treatment significantly reduced the percentage of apoptotic cells, as revealed by the TUNEL assay (Figure 1A). By staining cells with annexin V-FITC and PI, the percentage of viable, early apoptotic, late apoptotic, and necrotic cells were distinguished and quantitatively determined (Figure 1B). The ratio of healthy cells was higher in the presence of IGF-1 than in the control group. Compared with the none-stimulated cells, the proportions of early and late apoptotic cells were less in IGF-1 treated cells, respectively. In addition, IGF-1 triggered downregulation of the pro-apoptotic Bax protein amount and increased the anti-apoptotic Bcl-2 expression. Hence, IGF-1 visibly expanded the ratio of Bcl-2 and Bax in granulosa cells (Figure 1C).

The total amounts of Bim_EL_ protein declined distinctly in granulosa cells treated with IGF-1 (Figure 1B). At the same time, the reduced Bim_EL_ appeared to present diffuse phosphorylated bands on SDS-PAGE (Figure 1D). In order to verify whether the diffused bands were phosphorylated or not, lambda protein phosphatase (λ-PPase) was used. The results showed that the upper phosphorylated bands disappeared after the protein samples were subjected to λ-PPase digestion (Figure 1E). Conversely, the sample without the λ-PPase supplement still kept its primary phosphorylated and non-phosphorylated states (Figure 1E).

### 2.2. IGF-1-Induced Degradation of Bim_EL_ Regulated by ERK1/2 Pathway Was Associated with Proteasome Way

**Figure 2:**
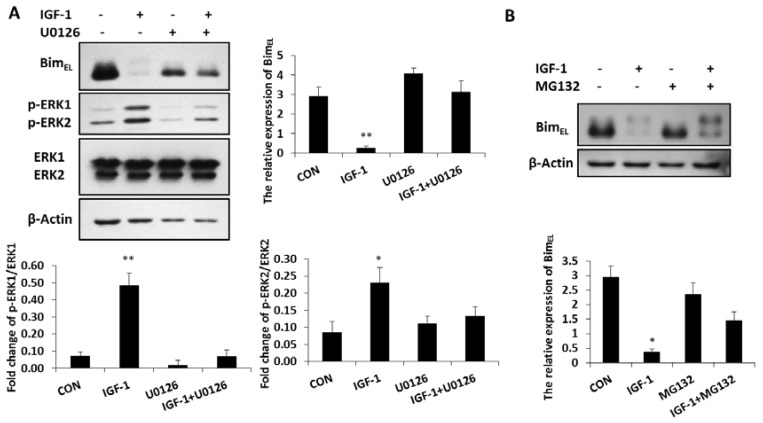
Inhibition of the ERK1/2 pathway impaired the effect of IGF-1 on Bim_EL_ and the proteasome system was related to Bim_EL_ downregulation. (**A**) Granulosa cells were treated with U0126 for 1 h before incubation in the presence of IGF-1 for 24 h. (**B**) Cells were pre-cultured in MG132 for 1 h and treatment with IGF-1 24 h. Bim_EL_, p-ERK1/2, ERK and β-Actin were detected with immunoblotting. Blots were probed with β-Actin to control for loading. Data are shown as means ± SD of at least three separate experiments. * *p* < 0.05, ** *p* < 0.01.

Bim_EL_ was phosphorylated and largely depleted in granulosa cells treated with IGF-1, while the concentration of phosphorylated ERK1/2 was significantly enhanced in these cells compared with that of the untreated group (Figure 2A). In the presence of the ERK1/2 pathway inhibitor, U0126, both phosphorylated ERK 1 and phosphorylated ERK 2 concentrations decreased, while the expression of Bim_EL_ was again up-regulated. However, when phosphorylated ERK1 and phosphorylated ERK 2 were inhibited by U0126, the ability of IGF-1 suppressing the Bim_EL_ protein decreased (Figure 2A). It showed that the reduction of Bim_EL_ resulting from IGF-1 was directly mediated by phosphorylated ERK1/2. The ubiquitin proteasome way plays a vital role in controlling protein turnover. Because IGF-1 promoted phosphorylation and downregulation of Bim_EL_ in Figure 1B,C, when the proteasome process was inhibited by MG132, the downregulation of Bim_EL_ stimulated by IGF-1 was restrained and the phosphorylation of Bim_EL_ increased (Figure 2B). Thus, the proteasome system was involved in the degradation of phosphorylated Bim_EL_ caused by IGF-1.

### 2.3. IGF-1 Induced Autophagy and Blocking Autophagy Flux Caused Accumulation of Phosphorylated Bim_EL_

**Figure 3:**
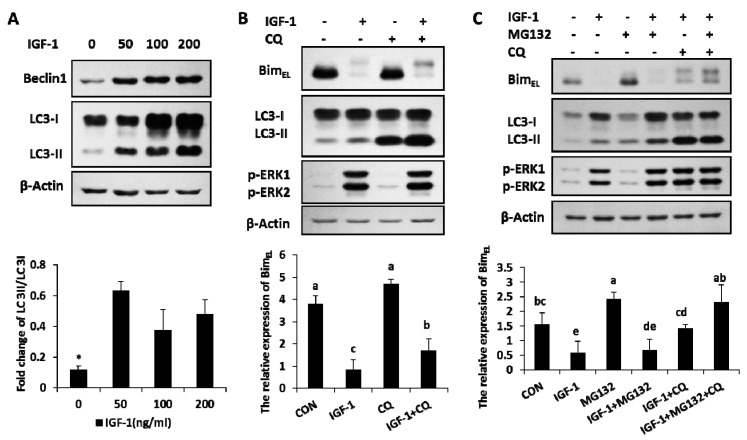
Autophagy was activated by IGF-1, and both blocking the autophagy flux turnover and inhibiting the proteasome system facilitated the accumulation of IGF-1-induced Bim_EL_ phosphorylation. (**A**) Beclin1 and LC3 were evoked in granulosa cells with different amounts of IGF-1. (**B**) The blockage of autophagy circulation accumulated the phosphorylation of Bim_EL_ in the presence of IGF-1. Cells were preconditioned with chloroquine (10 μM) for 1 h before the supply of IGF-1 in medium. (**C**) Both autophagy and proteasome processes were involved in the degradation of Bim_EL_. Cells were preconditioned with chloroquine (10 μM) and/or MG132 (5 μM) for 1 h before the addition of IGF-1. Normalized cell lysates were immunoblotted with antibodies for Beclin1, LC3, Bim_EL_, p-ERK1/2. β-Actin served as the loading control. The values are expressed as means ± SD of at least three separate experiments. * *p* < 0.05. a, b, c, d, e, different letters represent significant difference (*p* < 0.05) statistically, and same letters represent no change statistically.

The amount of Beclin1, the marker of autophagy, increased in the presence of IGF-1 with different concentrations (Figure 3A). The ratio of LC3-II, another autophagy marker, also enhanced with different levels of IGF-1 (Figure 3A). The results suggested that autophagy, an important cellular hydrolytic process, was induced by IGF-1. The relationship between Bim_EL_ expression and autophagy was further explored in another experiment. Granulosa cells were subjected to treatments of CQ (chloroquine), an autophagy flux blocker, to detect Bim_EL_ change. In the presence of CQ, the concentration of LC3-II had a definite gain, while the rate of LC3-II to LC3-I was significantly enhanced, which demonstrated that the autophagy flux was precisely blocked by CQ (Figure 3B). Compared to the single efficacy of IGF-1, after the blockage of autophagy turnover with CQ, the phosphorylated Bim_EL_ amount had a significant increase induced by IGF-1 (Figure 3B). In other words, blocking the autophagy flux turnover caused an accumulation of phosphorylated Bim_EL_ in the presence of IGF-1.

### 2.4. Both of Proteasome Way and Autophagy Were Involved in Bim_EL_ Degradation Induced by IGF-1

When both the proteasome system and autophagy were blocked by their inhibitors (IGF-1 + MG132 + CQ group), the IGF-1-induced Bim_EL_ degradation was completely blocked and restored the same concentration of Bim_EL_, similar to that of the untreated control (CON group). However, neither CQ (IGF-1 + CQ group) nor MG132 (IGF-1 + MG132) alone were able to achieve the complete blockage of Bim_EL_ degradation, suggesting that both the proteasome pathway and autophagy were involved in this process (Figure 3C).

## 3. Discussion

Ovarian oogenesis, follicle development, follicle selection and atresia are closely regulated by the crosstalk among cell survival and apoptosis signals, which include gonadotropins, steroids, cytokines and growth factors. Apoptosis presents in every stage of follicular development, but its regulations are stage-specific. During the primordial follicle stage, oocyte apoptosis is the major cause of follicle degeneration, and kit–kit ligand interaction most likely rescues the oocytes in fetal and postnatal ovaries [32]. In slow-growing pre-antral follicles, locally produced survival factors such as estrogen inhibit granulosa cells apoptosis [12]. During the normal reproductive cycle in animals, the antral stage is the critical period of follicle development and FSH is the major regulator at this stage [33]. In the progression to the pre-ovulatory follicles, both LH and gonadotrophins are responsible for follicle survival [34,35]. In antral follicles, IGF-1 is one potential crucial survival factors besides FSH [9]. As shown in the literature, in the antral stages, especially in the later stages, IGF-1 regulated the survival of cultured rat follicles [10]. In another study, although IGF-1 principally played important anti-apoptotic roles, IGF-1 stimulated the growth possibly by increasing the apoptosis rate and turnover of granulosa cells in healthy follicles, and in younger women rather than older women [13]. Furthermore, follicle development is closely associated with the development of granulosa cells [12]. In the present study, the mechanism of IGF-1 promoting degradation of Bim_EL_ to prevent granulosa cell apoptosis was investigated.

First, we found that IGF-1 inhibited granulosa cell apoptosis, which was associated with the downregulation of Bim_EL_ levels. Our previous reports showed that Bim_EL_ played a pivotal role in follicular atresia and granulosa cells apoptosis [30,36]. Bim (predominant isoform Bim_EL_), one pro-apoptotic BH3-only protein of the Bcl2 family, is required for the initiation of apoptosis induced by cytokine deprivation or stress stimulation [26]. T-ALL cells with low levels of Bim_EL_ are resistant to drug-induced death signals, but Bim_EL_ overexpression facilitates cell death [27]. Bim and other Bcl-2 family proteins are often regulated at the post-translational level through protein phosphorylation in leukemia cells, lung and colorectal cancer cells [37,38,39]. The phosphorylation of Bim_EL_ on serine 69 selectively leads to its degradation in K562 cells [40].

Next, in this study, it was validated that IGF-1 stimulated the phosphorylation of Bim_EL_ through a λ-phosphatase treatment trial. Thus, IGF-1 likely stimulated the downregulation of Bim_EL_ by promoting Bim_EL_ phosphorylation in granulosa cells. The numerous data indicate that multiple phosphorylation forms of Bim_EL_ are regulated by different MAPKs signal pathways [41]. JNK-mediated Bim_EL_ phosphorylation at S100, T112 and S114, caused by gliotoxin, effectively enhanced the stability and efficiency of Bim_EL_ [42]. Activation of p38 MAPK by sodium arsenite catalyzed phosphorylation of Bim_EL_ at serine 65, which promoted the apoptotic activity of Bim_EL_ in PC12 cells [43]. Inversely, the activation of the ERK1/2 pathway promoted phosphorylation and the reduction of Bim_EL_ in many cells, such as CCl39, CR1–11, and CM3 cells [44]. Previous reports have found that IGF-1 increased the degradation of Bim_EL_ protein by activation of ERK1/2 pathway in multiple myeloma [29]. Consistent with the above results, our results revealed that IGF-1 activated the ERK1/2 pathway to induce Bim_EL_ phosphorylation and promote its degradation in granulosa cells. This was verified because, after blocking the ERK1/2 pathway by U0126, the downregulation of Bim_EL_ stimulated by IGF-1 was abolished. The detailed phosphorylated sites of Bim_EL_ induced by IGF-1 are to be studied in the future. Other studies have shown that U0126 has inhibited other responses in porcine granulosa cells [45]. For example, in porcine granulosa cell line JC-410, the inhibition of ERK phosphorylation with U0126 blocked the IGF-1 induced activity of the IGF response element reporter gene from the P450scc promoter [46]. Additionally, MEK5 was shown to be partially inhibited by U0126 [47], and therefore the possible regulation of ERK5 to Bim_EL_ stimulated by IGF-1 should be further confirmed.

Third, the detailed degradation pathway of phosphorylated Bim_EL_ stimulated by IGF-1 was investigated. Our study showed that the subsequent degradation of Bim_EL_ was partially mediated through the proteasome system, as confirmed by the blockage of the 26S proteasome inhibitor, MG132. The ubiquitin–proteasome pathway was generally significant in the degradation of Bim_EL_ protein in various cells. [40]. For example, the toll-like receptor accelerated the degradation of phosphorylated Bim_EL_ through the proteasome system in mice immune cells [48]. Moreover, the non-harmful short-ischemic insult also caused Bim degradation, in the same manner, to reduce the damage of neurons, and when proteasome was blocked by MG132, the reduction of Bim was effectively inhibited [49]. Thus, this study indicated that the IGF-1 activating p-ERK1/2 stimulated degradation of Bim_EL_ was through the proteasome system in granulosa cells.

Moreover, in this study, autophagy was involved in the clearance process of phosphorylated Bim_EL_ induced by IGF-1. Autophagy circulation was induced by IGF-1 from our results. When autophagy flux was blocked by chloroquine, the degradation rate of Bim_EL_ slowed down, and phosphorylation of Bim_EL_ protein accumulated stimulated by IGF-1 in granulosa cells. Other researchers have also demonstrated that the autophagy pathway played a considerable role in the degradation of Bim_EL_, because autophagy downregulated pro-apoptotic Bim_EL_ in hepatocellular carcinoma cells, and the repression of autophagy reversed the decline of Bim_EL_ induced by low glucose and hypoxia [50]. In turn, Bim_EL_ consumption made autophagosome synthesis elevate in vivo, and that was inhibited by overexpression of the death-deficient Bim_EL_ [51]. Bim also blocked autophagy by interacting with Beclin1, an autophagy regulator [51]. Moreover, enhancing autophagy by IGF-1 from our study was consistent with a recent study showing that IGF-1 activated autophagy to prevent oxidant-induced apoptosis in vascular smooth muscle cells [52]. In addition, IGF-1 promoted the accumulation of autophagic vacuoles during glucose deprivation in rat cardiomyocyte-derived H9c2 cells [53]. By contrast, IGF-1 was also an important negative regulator of autophagy in some reports [54,55]. The knock-down IGF-1 markedly diminished the mTOR signal pathway and expanded autophagy production in bone marrow mesenchymal stem cells [56]. Therefore, IGF-1 acts as a double-edged sword in the regulation of the autophagy process. It possibly depends on the stimulation of the cell itself and the external environment, which can be fairly complicated. In this study, the blockage of both the proteasome and autophagy activities prevented Bim_EL_ degradation and increased accumulation of phosphorylated Bim_EL_ compared to blocking the one that was stimulated by IGF-1. Thus, both the proteasome and autophagy systems were involved in the degradation of phosphorylated Bim_EL_ induced by IGF-1.

**Figure 4:**
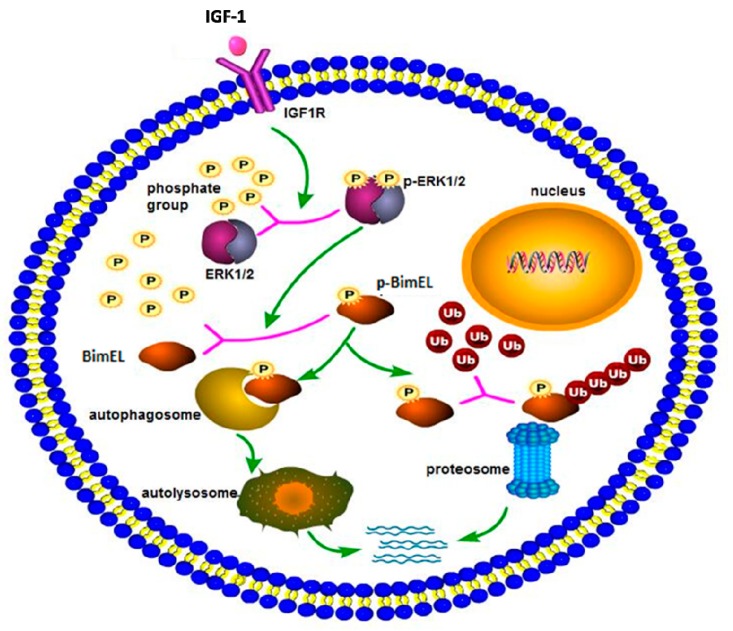
The schema diagram of IGF-1-induced Bim_EL_ degradation. IGF-1, combined with its receptor, spurred a series of downstream cascade reactions in porcine primary granulosa cells. IGF-1 activated the ERK1/2 pathway, which caused posttranslational phosphorylation modification of Bim_EL_. Modified Bim_EL_ was degraded by autophagy and the proteasome pathway, respectively.

In conclusion, a hypothetical model of Bim_EL_ degradation induced by IGF-1 is shown in Figure 4. IGF-1 can prevent porcine primary granulosa cells from apoptosis by regulating post-translation modification of Bim_EL_. First, IGF-1 induces the phosphorylation of Bim_EL_ through activating the ERK1/2 signal pathway. Then, the phosphorylated Bim_EL_ is degraded via both ubiquitin–proteasome and autophagy–lysosome pathways. However, the specific regulation of Bim_EL_ by IGF-1 is complex, and further work is required to elucidate the existing problems. Elucidation of the effect of IGF-1 on granulosa cell apoptosis can contribute to a better comprehension of follicular survival and atresia processes, and consequently, obtain increasing viable oocytes and embryos and improve in vitro assisted reproductive technology. Besides its applications in reproduction, the study regarding cell death can contribute to therapies for neoplastic and degenerative diseases.

## 4. Materials and Methods

### 4.1. Ethical Statement

All porcine sample collection procedures were performed in accordance with a protocol from the Animal Ethics Committee of the China Agricultural University (Permit Number: XK662, 6 April 2016).

### 4.2. Materials

Cell culture reagents were purchased from Invitrogen. Human insulin-like growth factor 1 (hIGF-1) was purchased from Cell Signaling Technology (Boston, MA, USA). Chloroquine and U0126 were from Promega Corporation (Madison, WI, USA). The MG132 was from Sigma-Aldrich (Louis, MO, USA). Lambda protein phosphatase (λ-PP) was from New England BioLabs Inc. (Ipswich, MA, USA). The following antibodies were used throughout whole study. Rabbit polyclonal antibody β-Actin (P30002, Abmart, China), rabbit polyclonal antibody LC3 (L7543, Sigma, USA), rabbit monoclonal antibody Bim (2933, Cell Signaling Technology, USA), rabbit polyclonal antibody phosphorylated ERK1/2 (9101, Cell Signaling Technology, USA), rabbit polyclonal antibody native ERK1/2 (9102, Cell Signaling Technology, USA), rabbit polyclonal antibody Beclin1 (B6061, sigma, USA), rabbit polyclonal antibody Bcl-2 (2870, Cell Signaling Technology, USA), and rabbit polyclonal antibody Bax (AF0057, Beyotime, China), as well as goat anti-rabbit second antibody, HRP (31460, Thermo Fisher Scientific, USA). All other chemicals were purchased from Sigma-Aldrich, unless otherwise stated.

### 4.3. Ovary Collection

Commercial Landrace × Large White pre-pubertal gilts aged approximately 6 months and weighing between 90 and 100 kg on average were used. The porcine ovaries isolated from pre-pubertal gilts were collected immediately after slaughter. Ovaries of well-developed in surface morphology and with no corpus luteum were selected and kept in a thermos bottle with 37 °C sterile physiology saline containing 100 IU/L penicillin and 100 mg/L streptomycin. They were transported to the laboratory within 2 h.

### 4.4. Cell Culture and Experimental Design

Ovaries were washed three times with sterile physiologic saline containing penicillin and streptomycin. Antral follicles, between 2 and 5 mm in diameter, on the surface of the ovaries were chosen. Porcine primary granulosa cells were cultured as previously described [57]. The granulosa cells were plated in 6-well plates at a density about 5 × 10^5^ cells/mL in the presence or absence of IGF-1 (50 ng/mL) for 24h. According to the following inhibitor experimental design, cells were pretreated with U0126 (10 µM), MG132 (5 µM) and chloroquine (10 µM) at 1 h before IGF-1 treatment. The doses and use of the selected drugs were from following the references [58,59,60,61]. Every experiment was conducted at least three times independently. Cells were counted with a hemocytometer and trypan blue staining (0.4% trypan blue in PBS), which showed that more than 90% of the cells were viable. Cells were incubated in a humidified 5% CO_2_ atmosphere incubator at 37 °C.

### 4.5. TUNEL Detection Assay

TUNEL trial was performed using the In Situ Cell Death Detection Kit (Roche Applied Science, Indianapolis, IN, USA) according to the manufacturer instructions. In brief, the cultured granulosa cells were washed thrice with 0.1% (w/v) PVA-PBS and fixed in 2% paraformaldehyde for 10 min. Cells were washed thrice and transferred to 0.1% Triton X-100 permeabilization solution at 4 °C for 10 min. Subsequently, cells were subjected to washing, incubating with fluorescein-conjugated dUTP and terminal deoxynucleotide transferase for 1 h at 37 °C in the dark in a humidified chamber. Nuclei were counterstained with Hoechst 33342 for 5 min and washed thrice. Samples were analyzed under a Leica fluorescence microscope. The excitation wavelength was in the range of 450–500 nm and detection was in the range of 515–565 nm.

### 4.6. Annexin V-FITC/Propidium Iodide Staining

After apoptotic stimulation, 0.1–1.0 × 10^6^ cells were harvested and washed twice with PBS. Cells were centrifuged at 500× *g* for 5 min at 4 °C and the supernatant was aspirated. Cells were resuspended in 195 µL staining buffer (Annexin V-FITC Apoptosis Detection Kit, Beyotime) and incubated with 5 µL of FITC-conjugated annexin V and 10 µL of propidium iodide (PI) for 15 min at room temperature in the dark. Samples were not stored, but analyzed immediately. Samples were analyzed with CytoFLEX flow cytometry (BECKMAN COULTER).

### 4.7. Western Blotting

The collected granulosa cell samples were washed with PBS and lysed in Laemmli sample buffer (Bio-Rad, Hercules, CA, USA). The equal amounts of protein (30μg) were electrophoresed and separated by sodium dodecyl sulfate polyacrylamide gel electrophoresis (SDS-PAGE, 12% acrylamide gel), and proteins were transferred to nitrocellulose membranes (Millipore, Billerica, MA, USA). After blocking with 5% non-fat milk in tris-buffered saline solution containing 0.1% tween-20 (TBST) at 37 °C for 1h, the membranes were incubated within primary rabbit polyclonal antibodies at 4 °C, overnight. Optimal dilutions for each antibody were determined (β-Actin, 1:2000; LC3, Bim, p-ERK1/2, ERK1/2, 1:1000; Beclin1, 1:500; Bcl-2, 1:1000; Bax, 1:500). Membranes were washed thrice with TBST solution and incubated with the appropriate secondary antibodies conjugated to horseradish peroxidase at a dilution of 1:2000 for 1h. Membranes were washed and protein bands were visualized using an enhanced chemiluminescence detection system (Applygen Technologies Inc., China). Images were taken and protein immunoblots were analyzed using ImageJ software (National Institutes of Health, Bethesda, MD, USA).

### 4.8. Lambda Phosphatase Treatment

Lambda protein phosphatase (λ-PPase) could remove the phosphate groups (dephosphorylate). In order to verify whether the diffused bands were phosphorylated or not, λ-PPase was used. Granulosa cells were cultured in the medium supplemented with IGF-1 for 24 h. Cells were collected, lysed and divided into two equal parts. One was treated with 1 µL λ-PPase (400U/µl, New England Biolabs, Beverly, MA, United Kingdom) at 30 °C for 2 h and the other was subjected to the same treatment, except for λ-PPase. After adding 10 µL 5× SDS sample buffer to two samples, samples were boiled for 10 min.

### 4.9. Statistical Analysis

Data are presented as mean ± standard deviation of at least three independent replicates. Data from assays were analyzed by one-way analysis of variance (ANOVA) and Duncan’s test using SAS software (SAS Institute, Cary, NC, USA). Significance was accepted at *p* < 0.05.

## Abbreviations

IGF-1	Insulin-like growth factor 1
Bim_EL_	Bcl-2 Interacting mediator of cell death-extra long
ERK1/2	Extracellular signal–regulated kinases 1 and 2
p-ERK1/2	Phosphorylated extracellular signal–regulated kinases 1 and 2
LC3	Microtubule-associated protein light chain 3
PI3K	Phosphatidylinositol 3-kinase
MAPK	Mitogen activated protein kinase
JNK	Jun N-terminal kinase
CQ	Chloroquine
λ-PPase	Lambda protein phosphatase
FSH	Follicle stimulating hormone
LH	Luteinizing hormone
